# Healthcare Resource Utilization for Chronic Rhinosinusitis in Older Adults

**DOI:** 10.3390/healthcare9070796

**Published:** 2021-06-25

**Authors:** David W. Jang, Hui-Jie Lee, Ryan J. Huang, Jeffrey Cheng, Ralph Abi Hachem, Chuck D. Scales

**Affiliations:** 1 Department of Head and Neck Surgery & Communication Sciences, Duke University, Durham, NC 27710, USA; jeffrey.cheng@duke.edu (J.C.); ralph.abi.hachem@duke.edu (R.A.H.); 2 Department of Biostatistics and Bioinformatics, Duke University, Durham, NC 27710, USA; hui-jie.lee@duke.edu; 3 Surgery Center for Outcomes Research, Duke University, Durham, NC 27710, USA; chuck.scales@duke.edu; 4 School of Medicine, Duke University, Durham, NC 27710, USA; ryan.j.huang@duke.edu; 5 Department of Surgery, Duke University, Durham, NC 27710, USA

**Keywords:** healthcare utilization, health services research, chronic rhinosinusitis, elderly

## Abstract

Objectives: Chronic rhinosinusitis (CRS) is a common and costly health problem in the United States. While often associated with younger adults, CRS can affect the elderly. As the aging population increases in the United States, the cost burden of CRS in older adults is important to assess. The objective of this study is to characterize healthcare resource utilization (HCRU) and healthcare expenditure (HCE) for CRS in this population. Methods: Patients meeting criteria for CRS with three years of continuous data were identified on IBM^®^ Marketscan Research Databases over a five-year period (2013–2017). Medication utilization, outpatient visits, surgery, and expenditures related to CRS were assessed for older adults (>65) and compared with other age groups. As a secondary analysis, multivariable generalized linear models were utilized to compare HCE while adjusting for baseline medication utilization. Results: A total of 238,825 patients met the inclusion criteria, of which 20,927 were older adults. Older adults had the highest overall prevalence of nasal polyps (10%) and asthma (16%) among adult groups. Surgery rate was lower than other adult groups, but medication utilization was the highest. Mean overall HCE at two years was highest in older adults (USD 2545 vs. 2298 in young adults). However, HCE was highest for the young adult group after adjusting for baseline medication usage. Conclusion: Older adults had a higher rate of CRS-related co-morbidities as well as the highest CRS-related medication utilization and unadjusted two-year HCE. Although the reasons for this are unclear, possibilities include greater disease severity and preference for medical versus surgical management. HCE for CRS is expected to increase as the aging population grows.

## 1. Introduction

Chronic rhinosinusitis (CRS) is one of the most common chronic conditions in the United States, with an estimated prevalence of 15% and annual direct costs of up to USD 9.9 billion dollars [1]. Although not life-threatening, CRS can have a significant impact on quality of life, with persistent symptoms and recurrent exacerbations despite medications and surgery. Due to the high economic burden of CRS, a closer examination into resource utilization for CRS is warranted. This would be critical in developing new strategies for cost reduction, quality improvement, and appropriate allocation of resources, especially as it pertains to government healthcare spending.

While CRS affects people of all ages, there is evidence that it may be more prevalent in older adults, as was reported by a population-based study from South Korea [2]. Other studies have suggested that older adults suffer from more severe CRS, as was demonstrated through endotyping and cluster analyses [3,4]. Others have demonstrated less improvement in quality of life scores after sinus surgery [5] as well as higher antibiotic utilization in older adults [6]. Finally, it is likely that older adults are less likely to undergo surgery due to co-morbidities, and therefore may need to tolerate prolonged periods with higher symptom burden.

It is estimated that 72 million Americans will be over the age of 65 by the year 2030 [7]. Therefore, the cost of healthcare for this population needs to be carefully assessed, especially as it pertains to chronic conditions such as CRS. However, there are very few studies investigating healthcare resource utilization (HCRU) and healthcare expenditure (HCE) for CRS in older adults from a population health perspective. Therefore, the aim of this study is to assess CRS-related HCRU and HCE in older adults, with the hypothesis that older adults account for a significant portion of resource utilization. Identifying this will allow for targeted measures to reduce cost and improve efficiency and quality of care in this population.

## 2. Methods

This study was approved by the institutional review board at Duke University (Pro00103732). This is a retrospective review of medical and pharmacy claims from the IBM Health MarketScan^®^ Research Databases over a five-year period (2013–2017). All patients (adult and pediatric) with continuous enrollment data between 1 January 2014 and 31 December 2015 were initially included. From there, patients with an index CRS diagnosis during that period were selected. Patients were considered as having CRS if CRS-related ICD diagnosis codes (ICD-9: 473.x, 471.x; ICD-10: J32.x, J33.x) appeared on at least two claims on two separate dates, as described by Rudmik et al. [8] From this cohort, patients with continuous one-year pre-index and two-year post-index enrollment were included. A one-year pre-index washout period was implemented to capture patients with a new CRS diagnosis. The index date was defined as the first date with a claim related to a CRS diagnosis, regardless of any provider in any setting. Claims on the index visits were used to identify demographic characteristics. One-year pre-index claims were analyzed to identify relevant co-morbidities. Patients with at least one claim of nasal polyps diagnosis (ICD-9: 471.x; ICD-10: J33.x) during the two-year follow-up were classified as nasal polyps subgroup. Appendix A (see Table A1) lists ICD codes associated with each comorbidity.

CRS-related HCRU and HCE per patient were determined for the two-year post-index period. HCRU included the following: CRS-related medication prescriptions, outpatient visits with an otolaryngologist, immunotherapy, and sinus surgery. HCEs were categorized into the following: CRS-related medications, CRS-related outpatient visits with an otolaryngologist, sinus surgery, and CRS-related overall cost. Overall cost included CRS-related medications, all inpatient and outpatient claims, office procedures, surgery, and imaging. CRS-related medications included any prescriptions in the following therapeutic classes: antibiotics, anti-inflammatory agents (includes nasal steroids), adrenals (include oral steroids), antihistamines, anticholinergics, mucolytics, sympathomimetics (decongestants), and leukotriene blockers. HCRU and HCE were then compared between four age groups: (1) pediatric (<18), (2) young adult (18–40), (3) middle adult (41–65), (4) older adult (>65).

Expenditures were based on paid adjudicated claims, including deductible, coinsurance/copayment, coordination of benefits and other savings (COB), and net payment (payment received by the provider excluding patient out-of-pocket and COB). Costs were adjusted for inflation based on US Bureau of Labor Statistics indices to 2017 USD. All costs reported were after excluding patients with capitated services and outliers (<1st percentile or >99th percentile of each cost). Patients with capitated insurance plans were excluded from the cost analysis.

Multivariable generalized linear models (GLM) using a gamma distribution with a log link were fit to compare the mean CRS-related costs over one-year and two-year periods between age groups, while adjusting for baseline pre-index medications. Cost ratios estimated by exponentiating the regression coefficients were reported with 95% confidence intervals (CIs). Cost ratios can be interpreted as a relative change in adjusted mean costs of one age category relative to a reference category (young adults). Regression-adjusted mean costs were calculated from the GLM to estimate the average marginal effects for each age group [9]. All statistical analyses were performed using SAS version 9.4 (SAS Institute, Cary, NC, USA) and R 4.0.0 (R Core Team, Vienna, Austria).

## 3. Results

**Figure 1:**
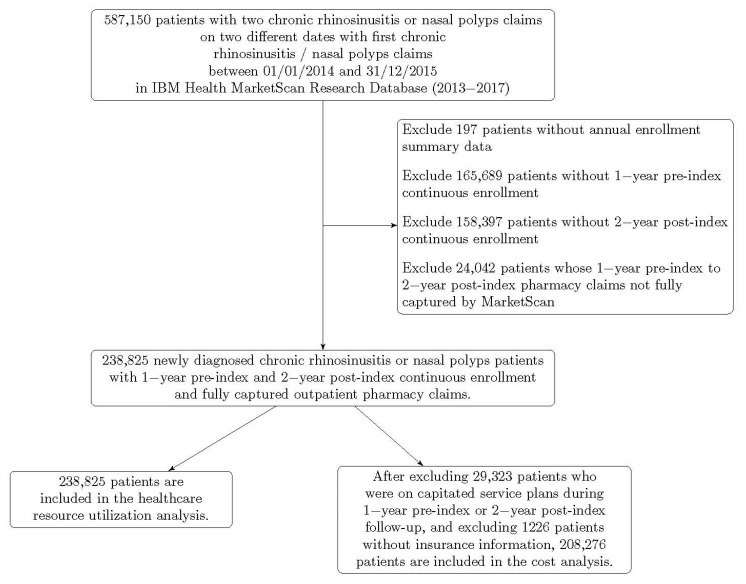
Cohort derivation.

There were 32,972,540 patients with continuous enrollment and complete claims data between 2014 and 2015. Of these, 238,825 unique patients met inclusion criteria. 20,927 patients were older adults. A total of 208,276 patients were included in the cost analysis after patients with capitated plans were excluded. Figure 1 displays how the study cohort was derived. Table 1 shows demographic characteristics for each age group. The older adult group had the highest prevalence of asthma among adult cohorts, as well as the highest prevalence of nasal polyps, diabetes, and reflux.

**Figure 2:**
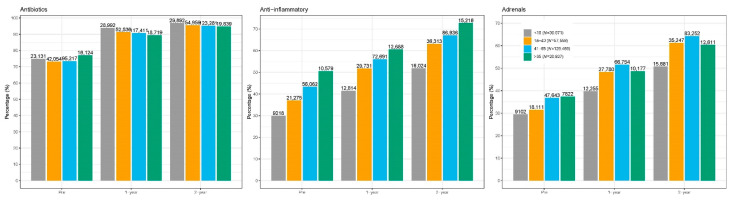
Medication utilization by age.

Two-year post-index HCRU is summarized in Table 2. Overall, antibiotics were the most commonly prescribed medication, followed by nasal steroids and oral steroids. Older adults had the highest prescription count for antibiotics among adults groups (average 6.5 prescriptions per patient), as well as the highest counts overall for nasal and oral steroids (Figure 2). Older adults also had the highest proportion of subjects with at least one visit to the otolaryngologist (54.5%). However, surgery rates were the lowest among the adult cohorts (8.2%).

**Figure 3:**
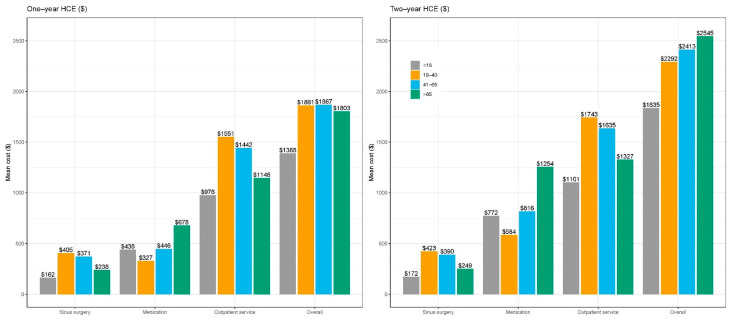
One and two-year HCE by age.

HCE at one-year and two-years after index diagnosis is summarized in Figure 3. Overall HCE one-year after index diagnosis was similar across adult cohorts ranging between USD 1800–1870 per patient. However, the older adult group had the highest overall HCE at two years at USD 2545 per patient. HCE due to medications was nearly two-fold higher that the other cohorts (USD 1253 vs. 816 in the middle adult group and USD 584 in the young adult group). In multivariable GLM (Table 3), the two-year adjusted HCE was highest in the young adult group, and lowest in the pediatric group, followed by the older adult group. Using the young adult group as the reference, the two-year HCE was 24.9% lower in the pediatric group (cost ratio 0.751, 95% CI 0.739 to 0.764), 2.8% lower in the middle-aged group (cost ratio 0.972, 95% CI 0.961 to 0.984), and 10.5% lower in the older adult group (cost ratio 0.895, 95% CI 0.878 to 0.912).

## 4. Discussion

The high economic burden of CRS combined with the need for improved healthcare efficiency calls for a close examination into how limited resources are utilized from a population health perspective [10,11]. Such an examination would allow for development of strategies to fairly allocate resources, reduce cost, eliminate waste, and at the same time, improve the quality of care. This is especially true for older adults, who often rely on government-sponsored healthcare in the United States. With the growing older adult population, assessing resource utilization for CRS and other common chronic conditions in older adults is an important endeavor.

We hypothesized that older adult patients would have higher HCRU and HCE based on recent studies that have investigated CRS characteristics and outcomes in elderly patients. An epidemiologic study of the Korean National Health and Nutrition Examination Survey revealed a higher incidence of CRS in the elderly population [2]. Smaller cohort studies using endotyping and cluster analyses have pointed to higher severity of CRS in older adults [3,4]. Other studies have demonstrated less improvement in sinonasal and general quality of life scores after sinus surgery [5] in addition to higher antibiotic utilization in older adults [6].

Consistent with these studies, our results suggest that older adults have more severe CRS than younger adults. The older adult group had a higher incidence of asthma and nasal polyps, and were prescribed greater numbers of antibiotics and oral steroids. Not surprisingly, this led to the highest HCE at two years. The lower surgery rate for older adults is of interest. Our data suggest that older adults are seeing the otolaryngologist at comparable rates as other groups, but may be foregoing surgery and continuing with long-term medical management because of a higher prevalence of systemic co-morbidities and greater risk with general anesthesia. This is demonstrated in the fact that HCE is roughly equivalent during the first year after CRS diagnosis, but HCE, particularly related to medications, becomes much higher during the second year after diagnosis. Our data demonstrate an inverse relationship between age, surgery rates, and HCE, supporting prior studies showing that continue medical management is often not the most cost-effective option [12,13].

Our regression-adjusted analysis, which adjusts for pre-index medication utilization, found that HCE was not higher in older adults. This can be explained in two ways. First, older adults may take antibiotics and anti-inflammatory medications for a host of chronic conditions, some of which may not be for CRS [14]. Second, older adults may begin treatment for chronic nasal symptoms before a diagnosis of CRS is formally made by a healthcare professional. Therefore, baseline costs are higher in this group. Conversely, adjusted HCE for younger adults may be higher because they are often diagnosed with CRS in a setting of otherwise good health and relatively recent onset of symptoms.

Because the high HCE associated with older adults resulted mainly from high medication utilization, reducing polypharmacy may be a strategy that can reduce costs [15]. Our data indicate that medications lacking evidence for use in CRS (e.g., decongestants, antihistamines), or are considered secondary treatments (e.g., leukotriene blockers) were commonly used. Routinely evaluating the efficacy of these medications may help to reduce unnecessary utilization. Another strategy may be to further promote antibiotic stewardship in this group, as has been described in the pediatric population [16]. By far, the most commonly prescribed medication was antibiotics. With increasing evidence that CRS is often not associated with bacterial infection, educating providers and patients on antibiotic stewardship may help to reduce costs and ineffective care. Finally, there is a need for additional data surrounding the optimization of older adults for surgical interventions in the operating room and the office.

Clearly, HCRU and HCE are complex issues at the heart of population health science. Age is only one of many contributing factors that have recently been investigated in the literature [17,18,19]. However, our study was not designed to determine if age in itself is a determinant of healthcare utilization for CRS. Rather, this study looks to assess the cost burden of CRS care in older adults relative to other age groups, which can in turn guide resource allocation and government spending. Future studies will need to investigate age in addition to other important population-based factors and determinants of health that influence healthcare utilization. This will allow for reduction of inefficiencies and cost, as well as identifying opportunities for improving outcomes for underserved populations. In addition, new advances in CRS care such as biologics and the increasing use of technologies such as balloon sinus dilation in the older population need to be taken into consideration in future studies [20].

Limitations of this study are those inherent in claims-based administrative databases. Detailed clinical data are not available, and inclusion criteria do not accurately capture all relevant patients. However, such databases can provide important information for studies involving population cohorts, and the same stringent criteria used for small cohort studies are impractical to apply. It is also important to note that the Marketscan databases contain information on elderly patients with Medicare Supplemental plans, which is only a portion of all Medicare patients. Therefore, the proportion of Marketscan CRS patients in the older adult group (8.8%) is not a reflection of the true proportion in the general older adult population. However, the Marketscan databases allow for a unique opportunity to compare healthcare utilization amongst different age groups. Despite these limitations, our findings establish the presence of variations and disparities in healthcare utilization according to age. Such variations, although seemingly small per patient, can translate to billions of dollars in expenditures for the U.S. population. Future studies will need to investigate the underlying reasons for such variations, and then assess ways to reduce inefficiencies should they exist.

## 5. Conclusions

Healthcare utilization and expenditure for CRS were substantially higher in older adults, despite the lower rates of sinus surgery. In particular, medication costs for CRS increased disproportionately in older adults during the second year after diagnosis. Greater CRS severity and preference for medical therapy over surgery may account for this discrepancy. HCE for CRS is expected to increase as the aging population grows. Additional research is needed in order to develop strategies for reducing expenditures and improving efficiency in care.

## Figures and Tables

**Table 1:** Patient characteristics.

Patient Characteristics					
	<18 (*N* = 30,871)	18–40 (*N* = 57,558)	41–65 (*N* = 129,469)	>65 (*N* = 20,927)	Total (*N* = 238,825)
Age, mean (SD)	9.7 (5.2)	30.9 (6.9)	52.6 (6.5)	74.0 (6.4)	43.7 (18.8)
Sex (% male)	15,632 (50.6%)	19,914 (34.6%)	47,442 (36.6%)	8716 (41.6%)	91,704 (38.4%)
Region					
Northeast	3899 (12.6%)	9303 (16.2%)	22,102 (17.1%)	5207 (24.9%)	40,511 (17.0%)
North Central	6585 (21.3%)	11,252 (19.5%)	26,191 (20.2%)	7103 (33.9%)	51,131 (21.4%)
South	15,885 (51.5%)	28,692 (49.8%)	63,394 (49.0%)	6684 (31.9%)	114,655 (48.0%)
West	4288 (13.9%)	8132 (14.1%)	17,341 (13.4%)	1911 (9.1%)	31,672 (13.3%)
Unknown Region	214 (0.7%)	179 (0.3%)	441 (0.3%)	22 (0.1%)	856 (0.4%)
Metropolitan statistical areas (%)					
Rural	4217 (13.7%)	7571 (13.2%)	18,932 (14.6%)	3264 (15.6%)	33,984 (14.2%)
Urban	26,483 (85.8%)	49,833 (86.6%)	110,149 (85.1%)	17,645 (84.3%)	204,110 (85.5%)
Unknown	171 (0.6%)	154 (0.3%)	388 (0.3%)	18 (0.1%)	731 (0.3%)
Insurance Type					
HMO	3097 (10.0%)	5630 (9.8%)	12,586 (9.7%)	1562 (7.5%)	22,875 (9.6%)
PPO/EPO	17,757 (57.5%)	34,164 (59.4%)	76,237 (58.9%)	9080 (43.4%)	137,238 (57.5%)
POS	1633 (5.3%)	3290 (5.7%)	7954 (6.1%)	649 (3.1%)	13,526 (5.7%)
CDHP/HDHP	5236 (17.0%)	8754 (15.2%)	17,027 (13.2%)	82 (0.4%)	31,099 (13.0%)
Comprehensive	452 (1.5%)	808 (1.4%)	5231 (4.0%)	9195 (43.9%)	15,686 (6.6%)
More than 1 type or no insurance info	2696 (8.7%)	4912 (8.5%)	10,434 (8.1%)	359 (1.7%)	18,401 (7.7%)
Comorbidities					
Nasal polyps	1025 (3.3%)	4414 (7.7%)	11,059 (8.5%)	2092 (10.0%)	18,590 (7.8%)
Asthma	6093 (19.7%)	6941 (12.1%)	17,495 (13.5%)	3343 (16.0%)	33,872 (14.2%)
Allergies	10,977 (35.6%)	18,536 (32.2%)	40,965 (31.6%)	5953 (28.4%)	76,431 (32.0%)
Diabetes	191 (0.6%)	1963 (3.4%)	16,854 (13.0%)	5595 (26.7%)	24,603 (10.3%)
Reflux	1675 (5.4%)	5710 (9.9%)	22,825 (17.6%)	5437 (26.0%)	35,647 (14.9%)
Headache	3799 (12.3%)	12,740 (22.1%)	24,938 (19.3%)	3330 (15.9%)	44,807 (18.8%)
Anxiety	1836 (5.9%)	8677 (15.1%)	16,556 (12.8%)	2537 (12.1%)	29,606 (12.4%)
Depression	954 (3.1%)	5280 (9.2%)	12,630 (9.8%)	2023 (9.7%)	20,887 (8.7%)

**Table 2:** Two-year HCRU by age group.

Two-Year HCRU by Age Group					
	<18 (*N* = 30,871)	18–40 (*N* = 57,558)	41–65 (*N* = 129,469)	>65 (*N* = 20,927)	Total (*N* = 238,825)
Prescription Count, Mean (SD)					
Antibiotics	6.5 (5.3)	5.6 (4.6)	5.9 (4.9)	6.5 (5.5)	6.0 (4.9)
Anti-inflammatory	1.4 (2.5)	2.0 (3.1)	2.8 (4.1)	3.5 (4.5)	2.5 (3.8)
Adrenals	1.8 (3.6)	1.9 (3.4)	2.6 (4.4)	3.0 (5.2)	2.4 (4.2)
Sympathomimetics	1.2 (2.5)	0.8 (2.3)	1.0 (2.9)	1.1 (2.9)	1.0 (2.7)
Leukotriene	1.4 (3.8)	1.0 (3.2)	1.3 (3.7)	1.2 (3.4)	1.2 (3.6)
Antihistamines	0.6 (2.1)	0.8 (2.4)	0.9 (2.7)	0.7 (2.4)	0.8 (2.6)
Otolaryngologic Care					
Outpatient Visits, Mean (SD)	1.7 (5.2)	2.6 (7.2)	2.8 (7.8)	2.7 (6.6)	2.6 (7.3)
Percentage of Patients with Visit	11,346 (36.8%)	27,663 (48.1%)	66,472 (51.3%)	11,411 (54.5%)	116,892 (48.9%)
Surgery Rate	1769 (5.7%)	7155 (12.4%)	14,458 (11.2%)	1709 (8.2%)	25,091 (10.5%)
Immunotherapy Rate	2159 (7.0%)	5034 (8.7%)	10,078 (7.8%)	949 (4.5%)	18,220 (7.6%)

**Table 3:** Regression-adjusted overall CRS-related costs per patient.

Age Group	Adjusted Mean 1-Year Cost (95% CI) ^1^	Cost Ratio at 1-Year (95% CI) ^1^	Adjusted Mean 2-Year Cost (95% CI) ^1^	Cost Ratio at 2-Year (95% CI) ^1^
Pediatric (<18 years old)	USD 1378 (1357, 1399)	0.687 (0.674, 0.700)	USD 1867 (1840, 1894)	0.724 (0.712, 0.736)
Young adults (18–40 years old)	USD 2006 (1983, 2030)	Reference	USD 2580 (2550, 2609)	Reference
Middle-aged adults (41–65 years old)	USD 1903 (1888, 1918)	0.949 (0.937, 0.961)	USD 2517 (2497, 2538)	0.976 (0.964, 0.988)
Older adults (>65 years old)	USD 1609 (1581, 1638)	0.802 (0.786, 0.820)	USD 2257 (2219, 2295)	0.875 (0.858, 0.893)

Note: All results in this table are the average of the marginal effects (AME) over the observations from GLM with a gamma distribution and log-link. *p*-values are all less than 0.0001. ^1^ Models adjusted for baseline CRS-related medication cost and age group at diagnosis.

## Appendix A

**Table A1:** ICD codes used to define comorbidities.

Description	Types of Codes	Codes
Diabetes	ICD-9-CM	250.0x–250.9x
	ICD-10-CM	E10.x, E11.x, E13.x
Asthma	ICD-9-CM	493.xx
	ICD-10-CM	J45.xx
Allergies	ICD-9-CM	477.xx
	ICD-10-CM	J30.x
Reflux	ICD-9-CM	530.11, 530.81
	ICD-10-CM	K21.x
Nasal Polyps	ICD-9-CM	471.x
	ICD-10-CM	J33.x
Headache	ICD-9-CM	307.81, 339.xx, 346.xx, 784.0
	ICD-10-CM	G34.xx, G44.xx, G50.0, R51
Anxiety	ICD-9-CM	293.84, 300.00, 300.01, 300.02, 300.09, 300.10, 300.20, 300.21, 300.22, 300.23, 300.29, 300.3, 300.5, 300.89, 300.9, 308.0, 308.1, 308.2, 308.3, 308.4, 308.9, 309.81, 313.0, 313.1, 313.21, 313.22, 313.3, 313.82, 313.83.
	ICD-10-CM	F06.4, F10.180, F10.280, F10.980, F12.180, F12.280, F12.980, F13.180, F13.280, F13.980, F14.180, F14.280, F14.980, F15.180, F15.280, F15.980, F16.180, F16.280, F16.980, F18.180, F18.280, F18.980, F19.180, F19.280, F19.980, F40.xx, F41.xx, F93.0, F94.0
Depression	ICD-9-CM	300.4, 301.12, 309.0, 309.1, 311
	ICD-10-CM	F32.0x, F32.1x, F32.2x, F32.3x, F32.8x, F32.81x, F32.89x, F32.9x, F33.0x, F33.1x, F33.2x, F33.3x, F33.8x, F33.9x, F34.1x, F43.21x
